# Molecular and Cellular Interactions of Allogenic and Autologus Mesenchymal Stem Cells with Innate and Acquired Immunity and Their Role in Regenerative Medicine

**Published:** 2017-01-01

**Authors:** Roghayeh Hosseinikia, Mohammad Reza Nikbakht, Ali Asghar Moghaddam, Ahmad Tajehmiri, Mahboobe Hosseinikia, Farhad Oubari, Mahin Nikougoftar Zarif, Yahya Pasdar, Kamran Mansouri

**Affiliations:** 1 Department of Health Education and Nutrition, Faculty of Public Health, Kermanshah University of Medical Sciences, Kermanshah, Iran; 2 Department of Pharmacology, Faculty of Medicine, Yasuj University of Medical Sciences, Yasuj, Iran; 3 College of Veterinary Medicine, Razi University, Kermanshah, Iran; 4 Medical Biology Research Center, Kermanshah University of Medical Sciences, Kermanshah, Iran; 5 Iranian Blood Transfusion Research Center, High Institute for Research and Education in Transfusion Medicine, Tehran, Iran

**Keywords:** MSCs, Immunomodulation, Cell therapy, Regeneration medicine, Soluble factors

## Abstract

Mesenchymal stem cells (MSCs), as major stem cells for cell therapy, have been studied from different aspects in preclinical and clinical settings for more than a decade. These cells modulate the immune system (humoral and cellular immune responses) in vitro by producing soluble factors (anti-inflammatory molecules) and/or making cell-cell contacts. Hence, they could be used in regenerative medicine, tissue engineering and immune therapy. MSCs-based therapy have been recently used for treatment of cancer regarding the migratory potential of these cells towards tumor cells which makes them considerable candidates, also for cell therapy in both allogeneic and autologous settings. So, this review attempts to focus on the factors secreted by MSCs such as cytokines, their functional role in mounting and controlling immune responses mediated by different immune cell subpopulations and their significance in regenerative medicine in clinical trials. Although, further studies remain to be done to increase our knowledge of regulating development mechanisms, homeostasis and tissue repair in order to provide new tools to implement the efficacy of cell therapy trials. Although MSCs have been proved safe and effective for cell therapy, there are still challenges to overcome before widely applying MSCs in clinic.

## Introduction

 Non-specific, self-renewal (long-term), multipotent stem cells are able to produce specific type of cells with specific functions. According to the definition, cell therapy is a subtype of regenerative medicine based on introducing a stem cell subtype in tissues without gene therapy.^1^ Recently, isolation of embryonic stem cells (ESCs) from inner cell mass (ICM) has provided a powerful tool for biological researches. Considering ethical limits on use of ESC and iPS in clinic, work field on MSCs have been prospering lately. It has demonstrated that bone marrow (BM) non-hematopoietic and fibroblast-like populations are able to participate in repairing of injured tissue.^2^ Also, Fridenstein et al. demonstrated that isolated different pluripotent populations from rodents bone marrow that have ability to form fibroblastoid colonies (CFU-F) when cultured on plastic, also they can participate in tissue repair and reconstitution of hematopoietic microenvironment when transplanted subcutaneously.^3^ Hence, the potential ability to culture cells and their differentiation into different mesenchymal lineages in vitro lead to enhance the use of them in tissue engineering and regenerative medicine. In addition, they called them MSC according to their ability of colony forming unit-fibroblast (CFU-F) and differentiation capacity.^4^^-^^6^ MSCs not only make mesodermal lineages such as chondrocytes and adipocytes but they also create endodermic and ectodermic cells. In experimental models, they have potential roles in tissue regeneration, angiogenesis, BM evolution, hematopoiesis and also in immunomodulation via producing a variety of soluble biological factors and making cell-cell contacts.^1^^,^^4^ By expressing of stromal derived factor-1 (SDF-1), MSCs can connect to CXCR4 expressed on surface of hematopoietic stem cells (HSCs). MSCs greatly participate in regenerating damaged tissues and also creating an appropriate feeder for HSCs in support of hematopoeisis and ESCs in vivo and in vitro. By having immunomodulatory effects, restoration potentials and reducing ethical concerns and teratoma formation, their application in clinic have been vastly increasing. There are a large number of growth factors released by MSCs including cytokines, chemokines involved in migration, expansion and factors involved in immunomodulation, angiogenesis and apoptosis.^6^^-^^8^ So, MSCs were confirmed to be effective in treating many diseases of immune and non-immune-based.^7^ Based on these studies, present article aims to discuss these properties and possible therapeutic perspectives regarding these cells. However, it has recently been observed that by transplantation of MSCs, inflammatory cytokines, ectopic tissue and neoplasm transformation are induced.^6^^,^^7^

**MSCs Immunobiology and Therapeutic Applications**

**Figure 1:**
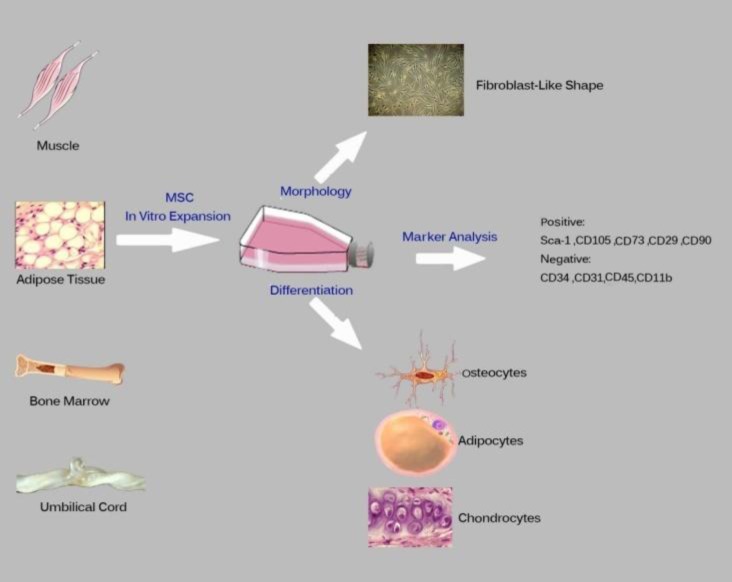
Properties of MSCs

In 1991, Simmons PJ et al. characterized MSCs surface antigens using STOR1^+^ antibodies.^9^ Earlier evidence showed that MSCs can exhibit phenotypic properties of endothelial, neural, smooth muscle, skeletal myoblasts and cardiac myocyte cells.^7^ According to International Society for Cell Therapy (ISCT), MSCs are defined as heterogeneous cells possessing: adhesive properties to plastic under standard culture conditions, Fibroblast–Like features, HLA-DR surface molecules in addition to ability to differentiate into trilineages in vitro, expression of CD105, CD73 and CD90, lack of expression of CD45, CD34, CD14 or CD11b and CD79a or CD19^6^^-^^8^(Figure 1).

Nowadays, in addition to BM, new sources have been introduced. MSCs are found in an anatomical location in around of vascular system of BM, isolated from HSCs, near endosteum.^7^^-^^10^ To obtain MSCs, various reports have addressed different sources of fetal and adult tissues with each exhibiting specific differentiation potentials.^11^ For example, osteogenic differentiation potency of placenta-isolated cells was lower than cells isolated from BM and that is due to the reduced expression of transcription factors Runx2 and Twist 2. While the higher expression of transcription factors involved in the early stages of chondrogenic and adipogenic differentiation such as PPARγ2 and SOX9 leads to higher levels of chondrogenic and adipogenic differentiating MSCs isolated from the placenta in comparison to those isolated from BM.^5^^,^^12^ Interestingly, some sources of MSCs such as amniotic membrane-MSCs (AM-MSCs) express CD45 known as HSCs marker. Two types of cells are isolated from fresh Aminiotic Membrane (AM) including Amniotic Membrane Endothelial Cell (AMEC) with central or eccentric nucleus and abundant vacuolated cytoplasm which are medium-sized round bi-nuclear cells expressing specific markers of ESCs such as stage specific embryonic Ag-3 and 4 (SSEA-3 and 4), tumor rejection Ag 1-60 (TRA1-60) and TRA 1-81, CD9, CD29, CD44, CD49d, CD44e, CD44f as well as molecules involved in cell adhesion and cell-cell contact. These cells exist in all three germ layers under ideal circumstances with limited number of them expressing CD45 marker. The other AM-isolated cell type, AM-MSCs, is a spindle-shaped adherent cell able to produce CFU-F and express similar markers to those of other MSCs sources and various expression levels of usual markers of MSCs (Figure 1).^13^

According to previous studies, undifferentiated MSCs have specific roles in modulating immune system.^7^^,^^13^ Therefore, frequent injections with HSCs, even as a graft, increase the success of tissue engraftment and decrease the risk of graft versus host disease (GVHD). Surprisingly, these cells could be grafted even in a recipient with incompatible HLA since they are already involved in hematopoiesis and participate in tissue repair. So, there is a worldwide overgrowing application of these cells in treatment of inflammatory and non-inflammatory diseases such as neurologic disorders, multiple sclerosis (MS), liver disease, lung damage,^6^^,^^12^ diabetes type I and II, duchenne muscular dystrophy (DMD), autoimmune arthritis, rheumatoid arthritis (RA), cardiovascular diseases, systemic lupus erythematosis (SLE), Alzheimer disease (AD), amyotrophic lateral sclerosis (ALS), GVHD, etc.^7^ Hence, these cells could be considered effective to increase cellular immune responses and protect against inflammatory reactions and infections.^14^^-^^16^ It is believed that MSCs are involved in growth, wound healing (See Table 1) and tissue repair. Based on previous studies, the transplantation of autologous BM-MSCs in liver diseases leads to improve clinical parameters of liver function in patients with liver cirrhosis or liver failure due to hepatitis B.^16^ MSCs also have potent therapeutic effects on skeletal muscle system and periodontal tissue healing, diabetes and skin defects after burn.^17^^-^^19^ Likewise, it has already been confirmed that MSCs effectively help with myocardial infarction (MI) treatment and corneal damages due to the release of tumor necrosis factor-inducible gene-6 protein (TSG-6) which in turn associates with modulating inflammation and tissue reconstruction.^1^^,^^4^^,^^6^

**Communication between MSCs and Immune System**

Nowadays, limited information is available on the genes and proteins engaged in MSCs functioning. Based on proteomic analysis and cDNA of MSCs and non-mesenchymal cells, despite the expression patterns of proteins and genes, fibroblasts and MSCs are a lot alike. Among several determinants such as growth factors, signaling cascades and extracellular matrix (ECM) factors controlling transcription, translation and cell count, those genes regulating surface markers and tumor genes could be noted as appropriate targets for therapeutic purposes.^1^^,^^7^^,^^8^

**Table 1:** Functional classes of wound healing proteins in human MSC-containing skin substitutes

**Specific Proteins**	**Primary Function**
MMP_1,2,3,8,10,13_	Matrix and Growth Factor Degradation, Facilitate Cell Migration
MMP_1,2_	Inhibit Angiogenesis and MMPs Activity
Ang-2, EGF, FGF-7 (Knows as KGF), PIGF, IGF, bFGF, PDGFs (AA,AB, BB), VEGFs (C, D)	Stimulate Migration and Growth, Promote Angiogenesis and Proliferative and Migration Stimulatory Effects
TGF-β3, HGF	Inhibit Scar and Contractive Formation
LIF	Support of Angiogenic Growth Factors
SDF-1β	Recruit Cell to Site of Tissue Damage

MSCs are pluripotent as ESCs since they express transcription factors such as Oct-4, Sox-2, Nanog and Rex-1.^7^^,^^8^ Modulatory role of allogeneic MSCs was first encountered and described where MSCs inhibited T cell proliferation both in vitro and in vivo. These cells are able to suppress immune system through intracellular interactions and releasing numerous bioactive soluble factors.^7^ Several surface and intracellular molecules are involved in inducing immunosuppression or any other changes in immune responses.^17^^,^^18^ Adhesion and MHC molecules play fundamental roles in interacting with immune cells, especially T lymphocytes, co-stimulatory molecules or Fas/FasL. When tolerance is induced, MSCs express low levels of MHC-I which are main components of antigen processing machinery (APM). They also reduce the expression of MHC-II, FasL and co-stimulatory molecules of B7-1 (CD80), B7-2 (CD86), CD40 or CD40L, while intracellular cell adhesion molecule-1 (ICAM1) is still expressed with induction.^20^ Those expressing Toll-like receptor 2, 3, 4, 7 and 9 have a specific role in modulating the immune system and have been affirmed that upon stimulation by TLR ligands, release anti-inflammatory modulators and subsequently modulate proliferation, differentiation, migration, survival and immune responses. MSCs lack programmed cell death-1 and 2 (PD-1 and 2) that are normally found on the surface of activated T and B lymphocytes and as well as interferon gamma (IFN-γ). However, IFN-γ-related increased expression of these molecules, negatively affects immunoglobulin like transcript receptor 2, 3, 4 (ILTR-2, 3, 4) in AM-MSCs.^8^^,^^13^^,^^19^ Immunomodulatory mechanisms of MSCs including I: inhibiting antigen presenting cells (APCs) activity through encountering T cells, II: suppressing immune system by inhibiting mediators such as prostaglandin E-2 (PGE-2), TGF-β, hepatocyte growth factor (HGF), IL-1β, Indoleamine 2,3 dioxygenase (IDO), hemeoxygenase-1 (HO-1), leukemia inhibitory factor (LIF), insulin-like growth factor (IGF), galectin, Jagged-1 and the isoform of HLA-G (sHLA-G5), III: up-regulating of internal pathway of IDO 2,3 expression, inducible nitric oxide synthase (iNOS) and HO-1. It is believed that cell-cell contact leads to the release of immunomodulators such as IDO, PGE-2, nitric oxide (NO) and HO-1 from immune cells, IV: inhibiting CD^4+^ and CD^8+^ cytotoxic activity, resting NK cells and producing regulatory cell populations in innate and adaptive immunity, V: up-regulating of IL-10 secretion by DCs and down-regulating IFN-γ, IL-2 or IL-15, VI: inhibiting B cell proliferative responses.^1^^,^^7^ It could be concluded that one unique feature of MSCs is that they express human non-classical human leukocyte antigen G (HLA-G) and co-stimulatory molecules such as B7-H1 and B7-H4 which negatively affect immune system.^20^^,^^21^ Expressing these antigens in normal and physiological conditions in testis, ovary and fetus cells (Immune-Privileged Organs) resembles an immunological tolerance developed through the activation of inhibitory receptors. On the other hand, MSCs with a fetal origin can increase the expression of HLA-G under influence of IFN-γ; so, they can be transplanted into a recipient of even incompatible HLA.^8^^,^^13^

**Key Role of MSCs in Immunomodulation**

During pregnancy, the immunological tolerance developed along with fetus development clarifies the key role of fetus-derived MSCs. These pleotropic cells inhibit a group of cells involved in innate and adaptive immunity such as B cell, DC, macrophages and various effector cells such as NK cells, T CD4^+^, T CD8^+^, Treg and NKT cells through complicated mechanisms identified both in vivo and in vitro. These cells are non-immunogenic since transplanting them into animals in vivo resulted in no immune response. This fact even makes it possible for these cells to be used in healing chronic wounds, burns and ocular disorders with no immunosuppressive medications needed.^13^^,^^22^ In addition to the important receptors involved in the migrating to damaged tissue like CCR1, CCR2, CCR4, CCR7, CXCR5, CCR10 and biological factors such as IL-6, LIF, stem cell factor (SCF), angiopoietin-1 (Ang-1) and Jagged-1, MSCs release suppressive factors such as IL-10, TGF-β, vascular endothelial growth factor (VEGF), soluble HLA-G (sHLA-G), keratinocyte growth factor (KGF), HGF, PDGF, IDO, NO and PGE-2.^23^^,^^24^ MSCs can even alter pattern of cytokine secretion from DCs, naive T cells, Th1 and Th2 due to mediating in the secretion of pro-inflammatory cytokines such as TNF-α (Tumor Necrosis Factor-α), IL-1β, IFN-γ and IL-10 (Tables 2, 3).^7^^,^^8^ According to Magatti et al. studies, AM-MSCs inhibited differentiation in monocytes and HSCs without making any cell-cell contact but through production of soluble factors such as cytokines and chemokines including CCL2, CCL8 and IL-6. It has been established that MSCs inhibit the secretion of inflammatory cytokines and chemokines such as TNF-α, CXCL10, CCL5 in cultured DCs.^25^^-^^26^

**Down-Regulatory Role of MSCs in Innate Immunity**

MSCs are capable of suppressing the responses of DC, macrophage and NK cell. In addition to induction of inhibitory cytokines, they can also play an important role in modulating the immune system due to their combined effects with NO, IDO, PGE2, TSG6, CCL2 and PD1.

**Table 2:** Summary of critical growth factor in tissue repair^10^

**Growth ** **Factors**	**Roles in MSC-Mediated Tissue Engineering**
EGF	Tissue Regeneration, Wound Healing and Neurogenesis
PDGF	Tissue Repair
FGF	Tissue Repair and Regeneration, Intrinsic Stem Cell Survival
TGF-β	Wound Healing
VEGF	Angiogenesis, Wound Healing
HGF	Vasculogenesis, Intrinsic Neural Cell Regeneration
IGF-1	Wound Healing, Neurogenesis
KGF	Wound Healing
Ang-1	Tissue Repair, Angiogenesis
SDF-1	Neuroprotective Effect, Wound Healing

**Table 3:** Summary of critical growth factor in immunomodulation^8^

**Soluble Factors**	**Function**
IDO	Inhibition of Proliferation
NO	Inhibition of Tryptophan
HGF	Inhibition of Proliferation, Cytotoxicity
sHLA-G, TGF-β	Inhibition of Proliferation, Cytotoxicity Promotion of Treg Generation
PGE-2	Inhibition of Proliferation, Cytotoxicity Stimulation of Cell Activation and Inhibition of DC and Treg Stimulation
IFN-γ, TNF-α, IL-1β	Promotes Chemokine Production and Immunosuppressive Factor Such as NO or IDO
IL-6	Regulates Migration, Stimulates Mitosis and Angiogenesis
IL-10	Inhibition of Apoptosis
VEGF	Inhibition of Apoptosis, Stimulates Angiogenesis
LIF	Inhibition of Apoptosis
SCF	Supports Growth and Differentiation
Jagged-1	Enhances Differentiation
CCLs, CXCLs	Promotes Migration of Leukocytes

These molecules were minimally expressed on the surface of inactive MSCs.^8^^,^^13^

**Dendritic Cells (DCs)**

DCs are the main cells in initiation, maintenance and regulation of immune responses in the antigen presentation process involved in innate immunity. During maturation, DCs express co-stimulatory molecules such as MHC-I, II and CD11c and CD83.^8^^,^^13^ MSCs have significant modulatory effects on APCs in innate immunity through:

1) blocking the monocyte maturation from CD14^+^ monocytes and CD34^+^ progenitor cells.

2) decreasing expression of co-stimulatory molecules such as CD40, CD80, CD83 and CD86, 3) inhibition of cell cycle, 4) preventing protein synthesis in stimulated monocytes.^8^ Also, impaired expression of CD1 in APCs in presence of MSCs^13^^,^^14^ may be resulted by PGE-2 and limited to early stages of DC maturation which is associated with changes in expression of DC surface markers such as CD80, CD83 and CD86 as well as IL-12 production.^23^^,^^24^ In presence of MSCs, DCs have:

i: lower levels of IL-12 and TNF-α production, ii: increase secretion of IL-1β and IL-10 and iii: reduce expression of MHC-II molecules.^8^ Recent studies have indicated that induction of DCs with HLA-G induces anergy and Treg cells induction. On the other hand, differentiation and maturation of monocytes into DCs in presence of MSCs (even using LPS)^25^^,^^26^ is impaired due to impaired allogeneic T cells stimulatory capacity. Furthermore, this condition is found to persist even after excluding MSCs and re-induction of monocytes which reflects irreversible functional changes made in monocyte differentiation by MSCs.^8^^,^^27^^-^^30^

**Natural Killer Cells (NK Cells-NKCs)**

NK cells act through cell surface receptors and stimulatory and inhibitory signal transductions. Depending on level of ligand and surface receptor expression on target cells and NK cells respectively, cytolysis of target cells can happen by NKCs.^4^^,^^31^^-^^33^ Through the secretion of immunosuppressive factors such as TGF-β, sHLA-G and PGE-2 as well as making cell-cell interactions, MSCs inhibit proliferation of IL-2 dependent cells.^8^^,^^10^^,^^34^ Moreover, MSCs inhibit cytotoxic activity of NKCs on virus-infected cells by reducing secretion of IFN-γ and consequently reducing NK cells response toward tumor and virus-infected cells. These reductions are results of reduced expression of NK cells receptors. According to Krampera et al. studies, attenuated NK cytolysis activity mostly occurs against HLA-I positive rather than negative type. Interaction between NKCs and MSCs not only induces anti-proliferative properties in NKCs but also activates those able to kill MSCs.^35^ Sometimes, MSCs can express ligands to actively bind to NK cells receptors, leading to increase NKCs activity and tumorcidal (cytotoxicity) properties.^7^^,^^8^ Nowadays, using reverse transcription polymerase chain reaction (RT-PCR) and enzyme-linked immunosorbant assay (ELISA) macrophage migration inhibitory factors and lytic activity inhibitor factors of NKCs can be quantified.^4^^,^^13^

**Role of MSCs in Acquired Immunity**

When T cell receptor (TCR) encounters an antigen, it proliferates while differentiating into effector cells releasing cytotoxic cytokines. It has been proved possible to inhibit proliferation of polyclonal mitogens stimulated T cells, allogeneic cells or specific antigens using AM-MSCs. It is also possible to inhibit stimulated PBMMNs with allogeneic cells or Phytohemagglutinin (PHA) through cell-cell interactions or Mixed Lymphocyte Reaction (MLR) approach and some cellular sources.^1^^,^^13^ By adding MSCs to MLR, Roelen et al. significantly increased mitogenic proliferative responses and production of important cytokines such as IL-2, IL-4, IL-7, IL-10, IL-15, IFN-γ (In secondary MLR) and VEGF during primary and secondary MLR. Also, similar studies were reported low levels of IL-17 and IFN-γ secretion in upper fluid of PBMNCs and AM-MSCs co-cultured in presence of mitogens compared to PBMNCs cultures alone.^28^ These researchers also detected high levels of IL-10 secretion and TGF-β in co-culture and increased expression of TGF-β, HGF, IDO and cyclooxygenase-2 (COX-2) mRNA. Immunosuppressive effect of MSCs was established in a Transwell system by adding IL-10 and TGF-β to MLR.^13^^,^^28^ Interestingly, fetal-derived MSCs exhibit stronger inhibitory effect on T cells due to greater production of IL-10 compared to maternal ones.^27^^,^^28^ Referring to former studies, expanded MSCs can have similar inhibitory effects on CD4^+^, CD8^+^ PB cells and expansion of CD4^+^ and CD8^+^ T cells of umbilical cord blood in response to PHA and PB lymphocytes. MSCs can suppress T CD4^+^, CD8^+^ cells and PBMNCs in Transwell system.^13^^,^^28^ Li C et al. established that placenta derived-MSCs (PD-MSCs) increase IL-10 (Th2 cytokine) level and decrease levels of IL-2 and IFN-γ (Th1 cytokines). Therefore, MSCs are able to change naive T path to either Th1 or Th2 cells.^29^ MSCs (particularly PD-MSCs) in addition to induction of IL-10 secretion, they also are able to activate T regulatory lymphocytes (CD4^+^, CD25^+ high^ and FOXP3 T Cells).^28^^,^^29^ Recently, 3-fold increase of T regulatory cells was noticed between co-cultured with placenta MSCs in comparison to isolated PHA. According to Li C et al. secreted factors by AMECs induce apoptosis inactivated T cells through activating Fas/Fas-L pathway via three mechanisms as follows: 1) hAM-MSCs are immunogenic and block production and maturation of APCs, 2) modulating cells and their proliferation 3) inducing inflammatory cytokine production.^29^

**T Cells and T Regulatory Cells**

**Figure 2:**
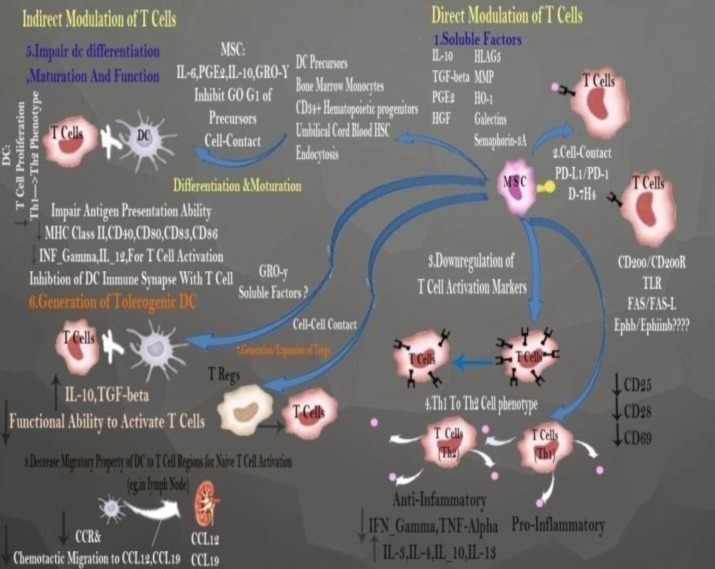
Direct and indirect mechanisms in modulating T cell responses

MSCs modulate T cells response through two mechanisms (Figure 2). MSCs inhibit mitogens activated T cell proliferation and T cell activated by specific antibodies against CD3 and CD28.^30^^-^^32^ MSCs negatively affect activation of markers such as CD25, CD38 and CD69. Therefore, these cells have similar effects on CD4^+^ and CD8^+^memory and naive T cells. In the presence of MSCs, T cells are arrested at the G0/G1 transition which depends on down regulating cycline D2 and reducing the expression of co-stimulatory molecules results in T cells anergy without apoptosis. Upon co-culturing MSCs with PBMNCs, an induced production of sHLA-G and CCL1 could be detected .This leads to a shift from allogeneic T cells toward Th2 response; so, cell lysis by allogeneic T cells can be inhibited.^7^^,^^8^ MSCs inhibit lymphocyte proliferation and immune response through triggering a denovo pathway and inducing proliferation of CD4^+^, CD25^+^ cells and Foxp3 and CD8^+^ regulatory T cells. 

By directly interacting with TCD4 cells and secreting PGE-2-and TGF-β, MSCs not only induce Treg cells but also specifically suppress T cell activation and proliferation in an indirect way by interfering with expression of HLA-G molecules and co-inhibitors such as B7-H4.^31^^-^^33^

**B Cells**

MSCs are able to 1) block proliferation of activated B cells in presence of IFN-γ, 2) negatively affect antibody production by B cells in a dose dependent manner through hMSCs, 3) inhibition secretion of chemokines involved in B cell migration. Although they do not affect co-stimulatory molecules and cytokine expression by B cells.^1^^,^^8^ Then, MSCs inhibit IgM, IgA and IgG production. The effects of MSCs on B cells are mediated by both soluble factors such as IFN-γ and IL-6 and also cell-cell contact.^34^^-^^38^

**Applications and New Approaches on Using MSCs in Clinic**

**Figure 3:**
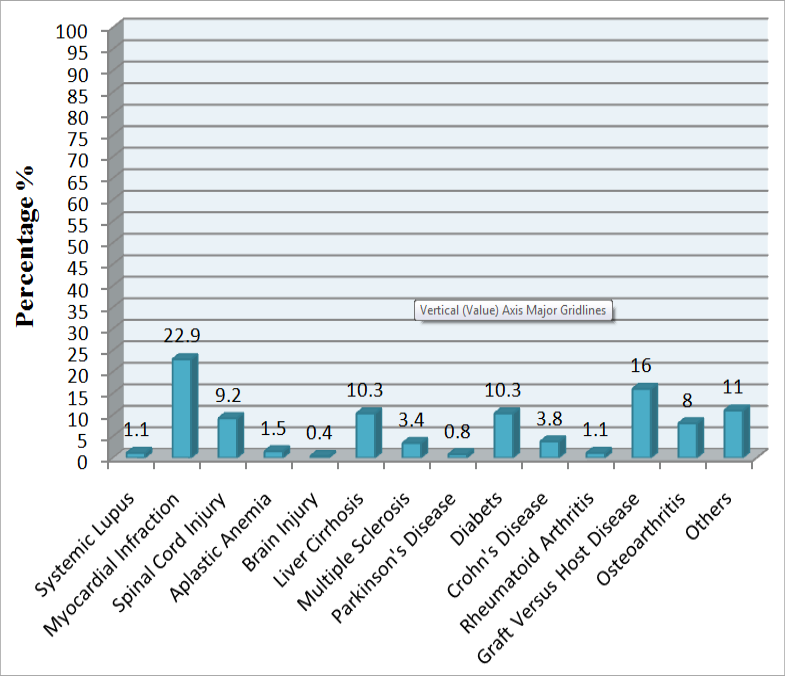
Percent of new common diseases treated with MSCs

**Figure 4:**
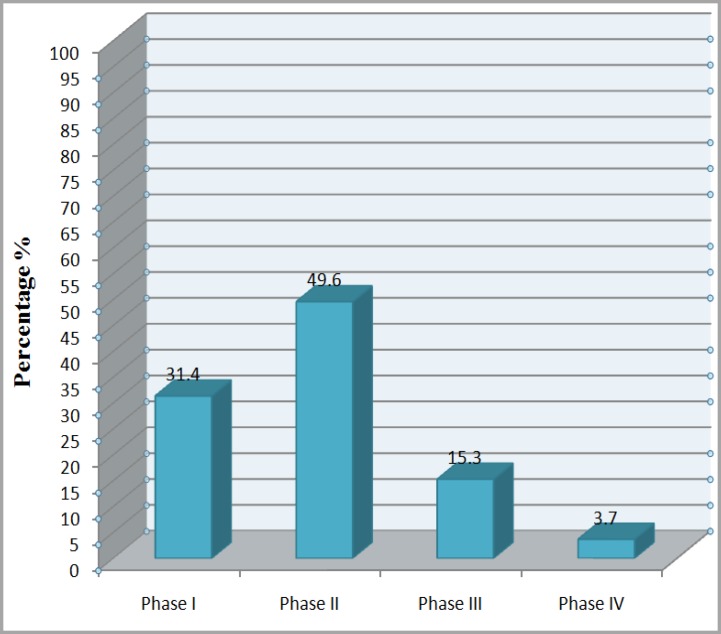
Phases of use of MSCs in cell therapy

**Figure 5:**
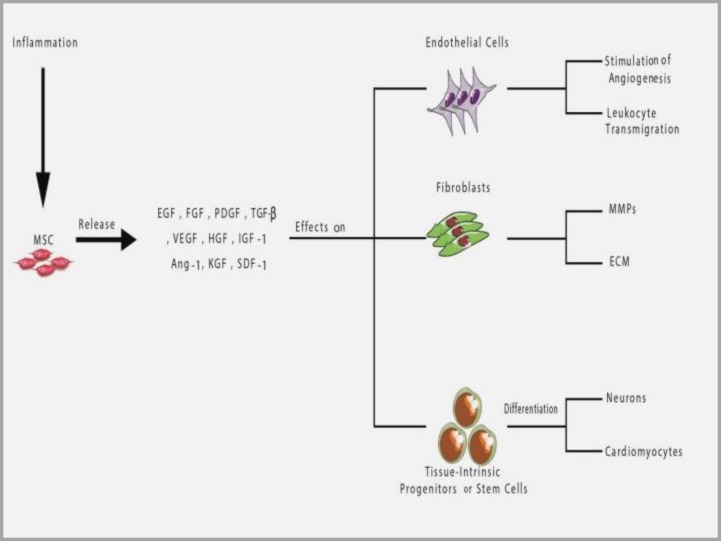
Tissue reparative properties of MSCs

MSCs are widely used in tissue engineering, regenerative medicine and preclinical phases of I-III (Figure 3 and 4).^34^^-^^36^ In vivo usage of MSCs are discussed as follows:

I:repairing damaged tissues without being rejected when transplanted into mice, II: injecting intravenous autologous MSCs with no following cytotoxicity induction, III: successfully developed HSCs transplantation resulting in reduced GVHD occurrence in patients and IV: reducing risk of acute and chronic GVHD in transplantation of allogeneic MSCs.^37^^-^^38^ Some reports of failure in preventing GVHD have also been issued on replacing BM and co-transplantation with HSCs, MSCs which caused BM stromal correction and improved grafted HSCs. It should be considered that MSCs are needed inflammatory environment for their activity. Therefore, monitoring of injection time, dosage and source of MSCs are necessary.^24^ MSCs promote tissue repair through migrating to damaged tissue. In 2000, by transplanting MSCs into the uterus of a sheep, long term graft survival extended to 13 months and due to efficient immunity conditions, grafted cells differentiated into chondrocytes, myocytes, adipocytes, cardiomyocytes, BM and thymoma stromal cells.^39^ Considering earlier studies, majority of transplanted cells are normally found in recipient's lung but gradually disappear.^40^ There are two approaches in functional systemic administration of MSCs: 1) according to migration potentials of MSCs in vivo and after being injected into tail vein (rat), they cause migration of cells into specific inflammatory tissues such as cartilage, liver and lung as transplanted cells survived to 13 months, 2) in 2007, following the injection into hepatic artery, an increased efficiency was observed in damaged tissues such as liver cirrhosis, osteonecrosis, skin disorders and spinal cord injury.^4^ Even systemic injection of stem cells in human and animal models resulted in an improved osteogenesis imperfecta (OI) and MI.^39^^,^^41^^,^^42^ Inflammation orients homing process of MSCs through molecules involved in cellular traffic such as chemockines, adhesion molecules and matrix metalloproteinase (MMPs). The most important chemokines, chemokine (C-X-C Motif) Ligand 12 (CXCL12 or SDF-1αβ), chemokine (C-X-C Motif) receptor 4 (CXCR4), Receptor (C-C Motif) Ligand 2 (CCL2 or MCP-1) and chemokine (C-C Motif) Receptor 2 are the central molecules involved in homing process.^43^ Expression of adhesion molecules VCAM-1 (CD106) and P-Selectin (CD62P) and interaction with very late antigen-4 (VLA-4) (CD49a, CD29) could be named as key mediator in MSCs rolling and induction of adhesion in vitro and in vivo. According to other studies, MSCs coated with antibodies against VCAM-1, lead to more efficient transplantation in colon and mesenteric lymph nodes in comparison to uncoated stem cells in a mouse model with inflammatory bowel disease.^44^ In addition to chemockines and adhesion molecules, most important MMPs, MMP-2 and MMP-membrane type 1, may also play a role in invasion of MSCs. The point here is that expression of molecules associated with homing could increase certain pro-inflammatory cytokines such as TNF and IL-1.^45^^,^^46^ In damaged tissues, MSCs release numerous growth factors to modify the tissue under influence of local stimuli such as inflammatory cytokines, TLR and hypoxia. Many of these factors are critical mediators of angiogenesis and apoptosis inhibitors such as VEGF, KGF, PDGF, IGF1, basic fibroblast growth factor (bFGF), HGF, IL-6 and CCL2 (Figure 5).^47^^,^^48^

By inducing tolerance in T cells and reducing pathogenic T and B cell immune response, MSCs are also effective in treatment of autoimmune diseases. A simple injection of MSCs in a model of collagen-induced arthritis prevents occurrence of an irreversible damage to cartilage and bone. PGE2-induced macrophages induce secretion of IL-10 from MSCs and prevent migration of neutrophils into tissues to effectively help with treatment of sepsis. The mechanism involved is that MSCs anti-inflammatory activity is induced by inflammatory macrophages.^49^^,^^50^ MSCs also have a specific role in treatment of pulmonary fibrosis, acute renal nephropathy, kidney damage, cancer, cartilage-bone diseases, and some other diseases.^4^^,^^13^ Through suppressing inflammation and decreasing damage in kidneys and intestines by inducing regulatory T cells, MSCs (autologous or allogeneic) play a significant role in treatment of patients with severe steroid resistance, SLE and Crohn's disease.^51^^-^^53^ Atrophy muscle syndrome (AMS)^54^ and stroke improvements have also been reported when MSCs were used as regulators.^55^

MSCs are capable of migrating to brain and cerebellum through blood-brain barrier, without destructing host brain architecture. In administration of allogeneic MSCs in experimental autoimmune encephalomyelitis (EAE) mouse model by infiltration of immune cells into spinal cord and decreasing levels of IFN-γ and IL-17, MSCs were associated with decreased score and severity of disease. MSCs act in central nervous system through cell replacement mechanisms and miming paracrine activity.^1^^,^^7^ Hence, in order to obtain meticulous understanding of subject, some restorative roles of MSCs are discussed as follows:

**Alzheimer Disease (AD)**

AD is known to be associated with 𝛽-amyloid deposition and neurofibrillary tangles formation which finally degrades cholinergic neurons. Recently, stem cell therapy has been used to study ameliorate neuropathological deficits in animal model of Alzheimer’s disease. Based on previous studies both ex vivo and in vivo, MSCs cleared amyloid plaque by evoking cell autophagy pathway and finally increased neuron survival.^49^ Moreover, some studies have shown that when adipose-derived MSC are transplanted in AD model mice, they harmonize inflammatory environment. This harmonization specially occurs by activating the microglia and elevating expression levels of alternative markers and 𝛽-degrading enzymes and decreasing expression levels of pro-inflammatory factors. Since treating autoimmune diseases with MSCs turned out to be promising, it was concluded that inflammatory environment of AD could also be modulated by MSCs. Since MSCs modulate microglial activation and AD patients suffer from Treg abnormalities of cell number and/or function.

Wei X et al. have published that transplanted umbilical cord-derived MSCs activated Tregs in AD animal model ex vivo and Tregs modulated microglia activation increasing neuron survival.^4^^,^^56^^,^^57^

**Asthma**

According to published studies, a reduced trachea of transplant-related bronchiolitis was found in rats treated with MSCs due to increased IL-10 and decreased TGF-β. Systemic injection of allogeneic MSCs increased IL-10 and IL-4 levels, decreased bronchial fluid and consequent induction of Treg and secretion of immunosuppressive molecules such as HGF and finally improved airway inflammation and hypersensitivity to protect the airway from IgE-mediated allergy. Therefore, allogeneic MSCs could be used in treat in respiratory diseases such as asthma.^4^^,^^7^^,^^13^

**Angiogenesis and Myogenesis**

Induction of angiogenesis and myogenesis by MSCs depend on the release of anti-apoptotic, myogenesis and angiogenesis factors such as VEGF, IGF-1, adernomedulin and HGF in diabetic patients. So, they are candidates for reducing cardiovascular complications in patients.^1^^,^^7^ On the other hand, due to the release of cardioprotectin factors from MSCs, angiogenesis and myogenesis were induced; so, cardiac dysfunction was improved. MSCs also help with treating and recovery of diabetic limb severe ischemia, bradycardia, reducing left ventricular pressure and contractility index and finally, increasing arterial pressure due to cardiac sympathetic nerve dysfunction in the diabetic animals. Studies showed that reducing serum glucose and insulin levels increases homing of transplanted cells in the pancreas and heart.^7^

**Anti Tumoral**

Tumors are considered as wounds that never heal or as an inflammation in which mobile MSCs are generated through secreting chemokines and cytokines.^57^ As a result, they frequently produce a variety of cytokines.^45^ The mobilized MSCs are able to migrate towards tumor and its surrounding tissues. According to this approach and involvement of tumor cytotoxic agents such as IFN-α, IFN-β, IL-12 and TNF-related apoptosis-inducing ligand (TRAIL) in human, MSCs have been a success in treatment of target tumors in animal models.^45^^-^^47^ Recently, MSCs have been studied as vehicles in delivery of nanoparticles to enhance effect of tumorcidals.^1^^,^^2^ In Zhang D et al. studies on rats, endostatin transferred with adenovirus (Ad-Endo) in mouse models, CPC was associated with migration to the tumor and angiogenesis inhibition.^58^ This study demonstrated the anti tumor properties with reduced tumor nodules, increased survival after transplantation, decreased proliferation, increased number of blood vessels and higher apoptosis rate were observed in tumors.^52^^,^^53^ In addition, using of photodynamic therapy in combination with MSCs as delivery vehicles of porphyrin loaded with nanoparticles has been proved innovative in osteosarcoma treatment.^54^^-^^56^ Where systemic treatments fail, MSCs help scientists come up with new ways of approaching different tumors including melanoma, medulloblastomas, pancreatic cancer and breast cancer and some other tumors.^52^

**Bone Repair**

Reconstructive role of allogeneic MSCs engineered with bone morphogenetic protein-2 (BMP-2) was studied in rats. Studied showed, genes transferred to MSCs contributed directly to bone repair while the remaining genes helped with reconstructing the damaged tissue.^1^^,^^7^ Thus, clinical benefits of allogeneic MSCs relate to their reconstructive and immunosuppressive properties. In the mentioned study, allogeneic MSCs loaded with Hydroxyapatite tricalcium Phosphate in dog femur led to the repair of segmental defect, with no further immunosuppressive therapy required.^7^^,^^13^

**Diabetes**

Considering the role of immunomodulatory and tissues repair by allogeneic MSCs, this source can be used as an appropriate candidate for treating diabetes type I and II. In diabetes type I, Islet cells are destroyed in an autoimmune process, whereas in diabetes type II, due to some functional imbalances, islet cells fail to control blood sugar.^54^^-^^56^ To treat the latter challenge, insulin and oral medications do not successfully control and prevent the complications associated with disease. Hence, alternative will be considered therapies to restore normal function of pancreas. According to studies, BM transplantation prevents destruction of Islet cells and in other clinical trials; MSCs led to successful transplantation of Islet cell in patients with type I diabetes. After inducing diabetes type I in rats with Streptozotocin and irradiating them to death, the injection of BM-MSC retrieved blood glucose and insulin levels to normal which indicates tissue repair in these animals.^1^^,^^7^^,^^8^ This condition is more effective when both types of cells (MSCs and Islet cells) were administered as a combination. MSCs are also able to help with repairing Islet cells and insulin-producing cells. These cells highly express genes associated with development of pancreatic beta cells and pancreatic duodenal such as Homebox 1, insulin and glucagon. Thus, these cells can release glucose-dependent insulin and recover Nuded Mice. Interestingly, hyperglycemia in vivo is considered as an important factor for BM-derived MSCs differentiation into cells producing insulin and normalizing glucose level in diabetic animal models. Therefore, Streptozotocin induced diabetic type I rats treated with MSCs exhibited decreased albuminuria and had normal histology image of glomerular.^1^ MSCs are able to repair necrotic fragments of diabetic kidney. Even in xenotransplantation, human beta cells into testis of rats, graft survived for only 17 days and was associated with insulin secretion, while in co-injection of beta cells and hMSCs into rats testis, engrafted cells were stable for 70 days and showed insulin secretion activity.^1^^,^^6^^,^^7^

**GVHD**

The first report on MSCs action as an immunosuppressive agent described a long-term survival of skin tissue transplantation. Co-injection of allogeneic MSCs and HSCs leads to an increased success rate in transplanting the tissue and preventing GVHD. Patients with transplanted HSC and non-responsive to steroid therapy were associated with shortened survival.^1^^,^^7^ Twenty eight days after injecting combination of steroids with Prochymal in patients with GVHD grade II-IV, approximately 93% of patients presented an initial response to Prochymal (77% of patients with a complete response and 16% partial response) without cytotoxicity and formation of ectopic tissue. It should be mentioned that Prochymal issued commonly in treatment of Crohn's disease and osteoarthritis.^1^^,^^7^ Interestingly, in a mouse model, MSCs failed to prevent GVHD, while in another study they prevented the occurrence of GVHD. 

**Hepatic Fibrosis**

Four weeks after injection of AMECs to liver fibrosis (mouse model) induced by carbon tetrachloride (CCL4), reduction of hepatic fibrosis was caused by activation of hepatic satellite cells of collagen producer, yielded a decrease in levels of hepatic proteins and profibrotic cytokines such as TGF-β1. Mice induction by CCL4 leads to T cell infiltration and increases the number of hepatic macrophages compared to a normal mice.^1^^,^^13^ Concurrent injection of MSCs with CCL4 leads to a further reduction in T cells, macrophages, chemokine and hepatic protein levels of macrophage chemo attractant protein-1 (MCP-1) in mice compared to CCL4 alone. In this study, increased expression of M2 macrophage-related genes such as YM-1, IL-10 and CD206 was detected which is related to fibrosis resolution and also hepatocytes were associated with reduced apoptosis, inflammation and fibrosis.^13^

**Intracerebral Hemorrhage**

Following MSCs transplantation, improvements of cerebral edema and correction of neurologic function were observed in rats with intracerebral hemorrhage. 28 days after MSCs transplantation into lateral ventricle in rats, animals behavior and brain edema were evaluated.^21^ Brain pieces were assessed in terms of morphology and immunohistochemistry by fluorescent microscopy. MSCs were evident in histological view along side wall and around damaged area and transplanted cells. These cells survived at least for 4 weeks. In these animals, microglial activation and water content of intracranial hemorrhage decreased and behavioral tests improved in comparison to control groups.^21^^,^^22^

**Ischemia**

With xenotransplantation of human MSCs in focal cerebral ischemia induced by artery occlusion in rat model, MSCs lead to improve function, reduce stroke volume and nerve protection granted to producing IGF-1, VEGF, epidermal growth factor (EGF) and bFGF (basic Fibroblast Groth Factor) in host brain. Even in a similar study, significant increase of nerutrophic factor and brain derived-neural growth factor (BD-NGF) with significant reduction of apoptotic cells were observed in ischemic areas. Neurons exposed to BD-NGF developed and boosted AKT pathway activity to protect them against withdrawal of trophic factors.^1^^,^^4^^,^^7^

**Lung Injury**

MSCs modulate inflammation and alleviate pulmonary fibrosis caused by bleomycin (mouse model) through reducing lung fibrosis and preventing pulmonary malfunction by lowering secretion of TNF-α, IFN-γ, MCP-1 and IL-6 in lungs. In this study, reduced infiltration of inflammatory cells and increased anti-inflammatory cytokines including IL-10 were observed. By MSCs transplantation, lung tissue was repaired through paracrine-like molecules and improved within 14 days in mice with induced lung fibrosis. MSCs also prevented development and distribution of fibrosis, proliferation of fibroblasts and lack of collagen replacement.^4^^,^^13^^,^^15^

**Multiple Sclerosis (MS)**

MS is an autoimmune disease of central nervous system and mostly depends on T cells. In this disorder, MSCs are involved in restoring both immunosuppressive and immunomodulatory features and also neural structures.^4^^,^^7^^,^^13^ Therapeutic effects of MSCs (AMEC) were studied in a murine model of experimental encephalomyelitis MS by injecting AMECs intraperitoneally in a mouse model of MS. Patel AN et al. noticed that symptoms were suppressed and demyelization was reduced. Axonal damage was also alleviated due to less proliferation of T cells and subsequent reduction in secretion of inflammatory cytokines.^7^ According to Li C et al. studies, MSCs injection lowers T CD3 cell counts, infiltration of monocytes/macrophage F4/80 (+) and CNS demyelization in a mouse model accompanying suppression of immune system mediated by production of PGE-2 and TGF-β. In this model, Th2 cytokine-dependent model and in particular, increased production of IL-5 was observed.^29^

**Parkinson’s Disease (PD)**

A neurodegenerative disorder associated with a progressive loss of dopaminergic neurons (DA), especially in parts compact of substantianigra. Several motor defects occur when mesostriatal dopaminergic pathway projected in striatum is absent. These defects include rigidity, bradykinesia and postural instability. Effective symptomatic therapy drugs like DA agonists of Levodopa (l-dopa) unfortunately associate with inefficient and some other side effects when used in long term. Replacing damaged neurons with stem cell therapy is yet the most promising strategy for disease.^56^^,^^57^

In PD animal models, MSCs proved to enhance levels of tyrosine hydroxylase and dopamine. Furthermore, these cells have been assumed to contribute to neuroprotection by secreting trophic factors like EGF, VEGF, NT3, FGF-2, HGF and BDNF or through antiapoptotic signaling without differentiation into neuronal phenotype. According to the obtained data, new strategies like modifying hMSCs genetically were suggested to induce the secretion of specific factors or to increase the percentage of DA cell differentiation.^56^

**Spinal Cord Injury**

MSCs have been used in treatment of animal models of this disorder in which inflammation was caused by secondary damage brings about lots of destruction effects. In case of spinal cord contusion model in monkeys without immunosuppression, transplanted cells managed to survive more than 120 days. Following transplantation, axons growth and formation of glial scar were detected and also death of neurons whose axotomized axons was prevented causing induction of new lateral spouts without inflammation and rejection in animals.^1^^,^^4^^,^^13^ Furthermore, corrections of locomotion functional tests in comparison to control animals were observed. Transplanted MSCs into rats with spinal cord injury led to an 8-week survival and it was observed that host spinal cord injuries were healed without being rejected compared to controls. Injected MSCs led to restoring axons, improvement of posterior limb function in rats and prevention of atrophy in cells with axotomized axons. Recently, it has been established that in brain of immunosuppressant rats transplanted with MSCs, hemisected are associated with microglial marker reduction and protein F4/80, indicating involvement of inflammatory cells in process of repairing spinal cord injury.^4^^,^^13^

**Stroke**

Inflammation is known as one of the main causes of cell secondary death following a stroke. MSCs obtained from various sources have novel therapeutic effects on eradicating inflammatory consequences. Therefore, after 1-2 days, direct transplantation of MSCs in Penumbra ischemic model improved functional disorders, associated neurological disorders and stroke size; depending on occlusion of cerebral stroke in comparison to control group which were injected with vehicle. 

Fourteen days after stroke and following the last behavioral test, histology was studied with Nissel staining and an increase of healthy cells was shrewdly observed in Penumbra Ischemic compared to control group injected with vehicle alone (Table 4).^55^

**New Required Protocol in Cell Therapy**

Regardless of all advances in cell therapy, it is still crucial to concern about following items:

1) Safety: Injected cells need to be non-toxic and associate with no long-term side effects. On the other hand, with over increasing tumor and metastasis rate, standard protocol must be considered. Insausti et al. observed that due to IL-6 secretion, MSCs worsen the arthritis in collagen-induced arthritis model caused by Th-17 increased activity.^13^ 2) Quality check: Injected cells must be germ free. Thus, lots of microbial tests need to be done during various stages of cell preparation. Other tests including viability, phenotype, oncogenicity and endotoxin evaluation tests also must be carried out. 3) Clinical grade production: since for using MSCs clinically numerous cells are needed, culturing these cells in vitro is inevitable. However, over passaging cells results in transforming them as Morini MR et al. illustrated this fact during a 4-5 month culture.^57^ Transformed cells contained chromosome changes, increased c-myc level and telomerase activity which finally led them to become tumoral. Therefore, preventing cells from aging and transformation by limiting number of passages is really vital for process.^53^^-^^57^

**Table 4:** Summary of clinical trials ^49^

**Studies**	**Origin**	**Route**	**Leads**	**Results**
Gang et al	UC-MSC	Transplantation	Skeletal Muscle Differentiation	MyoD, Myognin Expression in Mice Dystrophic
Shake and Nagay	A-MSC	Systemic Injection	Increase of Capillary, Decrease of Collagen and Fiber	Improvement of Infracted Myocardium in Rodent Model
Katritis et al	Autologus-MSC	Transplantation	Partial Improvement of Myocardial Contractility	-
Van Poll et al	A-MSC	Systemic Injection	Hepatocellular Death was Drastically Reduced, Hepatocyte Proliferation Increased	An Amelioration of the pathological Phenotype in a Rate Model for FHF
Gonzalez et al	AD-MSC	Systemic Injection	Decrease of Inflammatory Cytokines and Increase of Chemokines Associated to Th1/Th2,IL-10 and T reg	Migration to Joint (Anti-Inflammatory Response)
Zheng et al	MSCs isolated from RA Patients	Transplantation	Blooking the Secretion of Several Proinflammatory Cytokines and The Expression of Anti-inflammatory IL-10	Suppress of The Inflammation with Regulating The Secretion of TGF-β1 and Prevent of Joints Destruction
Sun et al	BM-MSC	Systemic Injection	Correlation of Hematological Disorders in SLE Patients	-

Although Bernardo et al. successfully passaged healthy MSCs up to 25 times.^34^ 4) Due the low level of MHC I, II and co-stimulatory molecules like CD40, CD80 and CD86 presentation, allogeneic and autologous MSCs are immune privileged making them good candidate to be used for allogeneic transplantation without being rejected.

5) Clinical transport: biologists and specialists need to gather in order to decide proper and concise rules considering standards for culturing, safety, administration time and location for MSCs. Hence to optimize clinical use of these cells and prevent possible side effects, lots of studies need to be carried out to finally revolutionize both immunologic and non-immunologic diseases treatment.^55^^-^^57^

## CONCLUSION

 MSCs have recently presented a new perspective to treat inflammatory and non-inflammatory diseases since they escape immune system by producing soluble factors (inducer or inhibitor) and molecules implicated with immune system and tissue repair. Meanwhile, regarding active transcription factors and differentiation potentials of them, using these cells require a certain criteria and standard (regarding culturing, differentiation, preparing quality and safety checks) in vitro and in vivo which are not available, yet. Against all these problems, vast applications of this cell source in regenerative medicine and other branches is attracting more attention daily and they are considered as candidates of delivering common drugs to tumors and malignancies in a more determined developed manner.
